# Monitoring Insect Resistance to Bt Maize in the European Union: Update, Challenges, and Future Prospects

**DOI:** 10.1093/jee/toac154

**Published:** 2023-01-04

**Authors:** Matías García, Carlos García-Benítez, Félix Ortego, Gema P Farinós

**Affiliations:** Laboratory of Applied Entomology for Human and Plant Health, Centro de Investigaciones Biológicas Margarita Salas, CSIC, 28040 Madrid, Spain; Laboratory of Applied Entomology for Human and Plant Health, Centro de Investigaciones Biológicas Margarita Salas, CSIC, 28040 Madrid, Spain; Laboratory of Applied Entomology for Human and Plant Health, Centro de Investigaciones Biológicas Margarita Salas, CSIC, 28040 Madrid, Spain; Laboratory of Applied Entomology for Human and Plant Health, Centro de Investigaciones Biológicas Margarita Salas, CSIC, 28040 Madrid, Spain

**Keywords:** insect resistance management, Bt maize, Cry1Ab, corn borers, MON810

## Abstract

Transgenic maize producing the Cry1Ab toxin of *Bacillus thuringiensis* (Bt maize) was approved for cultivation in the European Union (EU) in 1998 to control the corn borers *Sesamia nonagrioides* (Lefèbvre) and *Ostrinia nubilalis* (Hübner). In the EU since then, Cry1Ab is the only Bt toxin produced by Bt maize and Spain is the only country where Bt maize has been planted every year. In 2021, about 100,000 hectares of Bt maize producing Cry1Ab were cultivated in the EU, with Spain accounting for 96% and Portugal 4% of this area. In both countries, Bt maize represented less than 25% of all maize planted in 2021, with a maximum regional adoption of 64% Bt maize in northeastern Spain. Insect resistance management based on the high-dose/refuge strategy has been implemented in the EU since 1998. This has been accompanied by monitoring to enable early detection of resistance. The monitoring data from laboratory bioassays show no decrease in susceptibility to Cry1Ab had occurred in either pest as of 2021. Also, control failures have not been reported, confirming that Bt maize producing Cry1Ab remains effective against both pests. Conditions in the EU preventing approval of new genetically modified crops, including maize producing two or more Bt toxins targeting corn borers, may limit the future effectiveness of resistance management strategies.

## Resumen

El maíz transgénico que expresa la toxina Cry1Ab de *Bacillus thuringiensis* (maíz Bt) fue aprobado para su cultivo en la Unión Europea (UE) en 1998 para el control de los taladros del maíz *Sesamia nonagrioides* (Lefèbvre) y *Ostrinia nubilalis* (Hübner). Desde entonces en la UE sólo se ha sembrado maíz Bt que produce la toxina Cry1Ab, y España es el único país donde se cultiva ininterrumpidamente desde 1998. En 2021 se cultivaron en la UE unas 100.000 hectáreas de maíz Bt que produce Cry1Ab, de las cuales el 96% corresponde a España y el 4% a Portugal. En ambos países, el maíz Bt representó menos del 25% de todo el maíz sembrado en 2021, con una adopción regional máxima del 64% de maíz Bt en el noreste de España. El manejo de la resistencia de insectos, basado en la estrategia dosis-alta/refugio se ha aplicado en la UE desde 1998. Esto ha ido acompañado de un seguimiento que permite la detección temprana de la resistencia. Los resultados del seguimiento a partir de bioensayos de laboratorio muestran que hasta 2021 no se ha producido una disminución de la susceptibilidad a Cry1Ab en ninguna de estas plagas. Además no se han registrado fallos en el control, confirmándose que el maíz Bt que produce Cry1Ab sigue siendo eficaz frente a ambas plagas. Las condiciones de la UE que impiden la aprobación de nuevos cultivos genéticamente modificados, incluido el maíz Bt que produce dos o más toxinas para el control de los taladros, puede reducir la eficacia futura de las estrategias de manejo de la resistencia.

## Current Status of Bt Maize Cultivation in the EU

The cultivation of genetically modified (GM) maize producing insecticidal proteins from the bacterium *Bacillus thuringiensis* (Bt maize) has grown exponentially since the first varieties producing the Cry1Ab toxin were used in 1996 in the United States and a year later in Canada to control the European corn borer, *Ostrinia nubilalis* (Hübner) (Lepidoptera: Crambidae), and the Southwestern corn borer, *Diatraea grandiosella* Dyar (Lepidoptera: Crambidae), respectively (Buntin et al. 2004). So far, a total of 210 GM maize events conferring insect resistance (181 against Lepidoptera and 126 against Coleoptera) have been developed for commercialization (ISAAA 2019, 2022). Bt maize has been widely cultivated in many countries due to its efficacy against some harmful pests, which makes Bt maize competitive from an agronomic point of view (Dively et al. 2018, Marques et al. 2019) in addition to providing economic, environmental and social benefits (Sanahuja et al. 2011, Brookes 2019, Ala-Kokko et al. 2021). In the European Union (EU), the importation of maize grain from 159 GM maize events producing different insecticidal toxins is authorized for the placing on the market of products containing, consisting of, or produced from this maize (European Commission 2022a, ISAAA 2022). However, only two of these events, both producing Cry1Ab, have been authorized for cultivation to date: Bt176 (Syngenta) from1998 to 2005, and MON810 (Monsanto) since 2003. Maize hybrids derived from event Bt176 were withdrawn from the European market in 2006 because this event contained an ampicillin resistance gene as selectable marker (Badosa et al. 2004, Devos et al. 2006). Therefore, since 2006 only one GM plant, MON810 maize, has been commercially grown in the EU. From 2011 to 2021 it was grown on ca.100,000–130,000 ha annually, accounting for 0.05% of the world area of GM crops in 2019 (ISAAA 2019, DGAV 2022, MAPA 2022a).

**Fig. 1.**
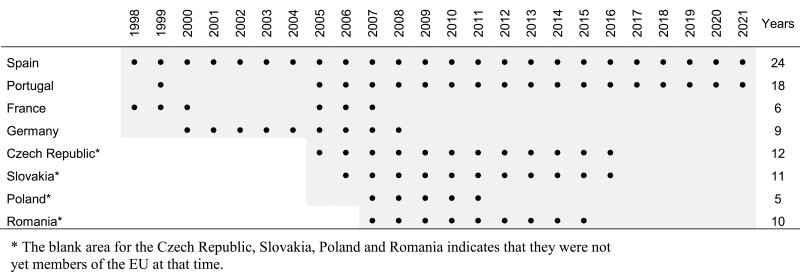
European Union countries that have grown Bt maize (•) (Bt176 and/or MON810 events) since its release in 1998. Sources: Annual Briefs from the International Service for the Acquisition of Agri-Biotech Applications (ISAAA) (https://www.isaaa.org/resources/publications/briefs/default.asp, accessed: 1 July 2022), MAPA (2022a), and DGAV (2022).

Bt176 and MON810 produce the lepidopteran-specific insecticidal toxin Cry1Ab (Van Frankenhuyzen 2009, Pardo-Lopez et al. 2013). The cultivation of these Bt maize hybrids in Europe was initially intended for the control of *O. nubilalis*, but they were also shown to effectively control another key pest of maize in the Mediterranean area, the Mediterranean corn borer *Sesamia nonagrioides* (Lefèbvre) (Lepidoptera: Noctuidae) (González-Núñez et al. 2000, Eizaguirre et al. 2006). Of the 27 countries that currently make up the European Union, eight have grown Bt maize at some point: Spain, Portugal, France, Germany, Czech Republic, Slovakia, Poland, and Romania. However, since 2017 only Spain and Portugal have planted this crop and of these, only Spain has grown it continuously since 1998 (Fig. 1).

**Fig. 2.**
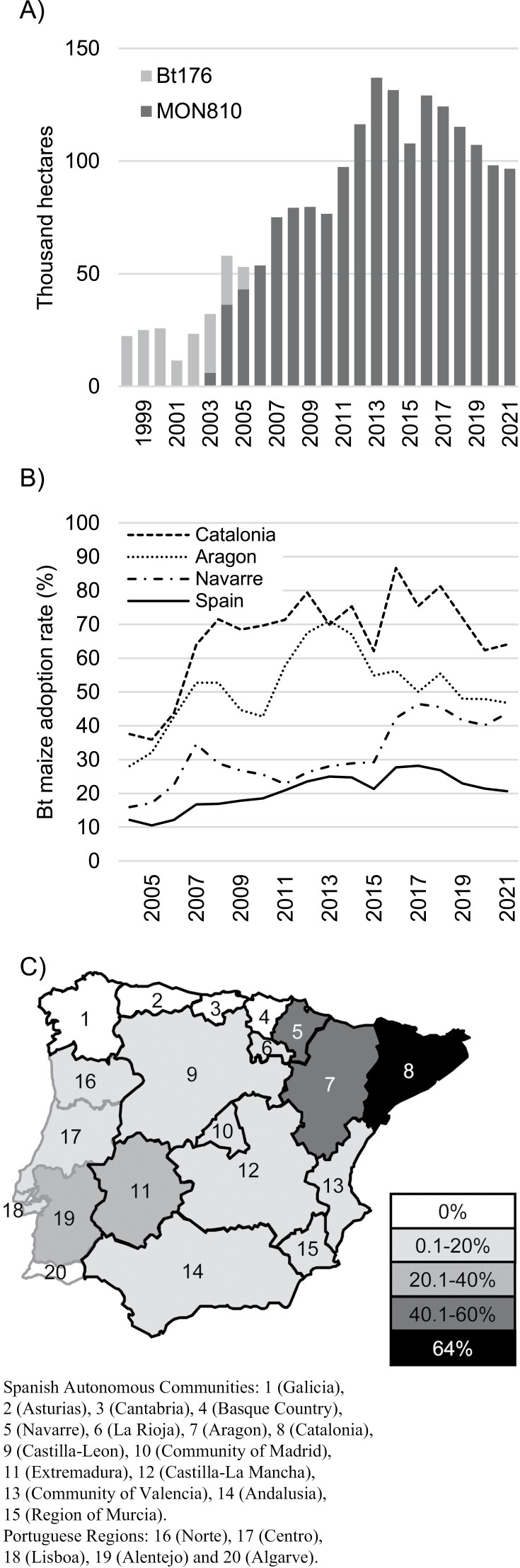
Bt maize in the Iberian Peninsula (Spain and Portugal). (A) Area of Bt maize (Bt176 and MON810) in Spain from its introduction in 1998 to 2021. (B) Bt maize adoption rate in Spain and the three Autonomous Communities in NE Spain: Aragon, Catalonia and Navarre. (C) Bt maize adoption rate per area in the Iberian Peninsula in 2021. Sources: MAPA 2022a, b, DGAV 2022.

The area under Bt maize cultivation in Spain has been gradually increasing to reach about 100,000–125,000 hectares since 2011 (Fig. 2A) (MAPA 2022a). Currently, it represents 96% of the GM maize grown in the EU. The remaining 4% is grown in Portugal, where MON810 varieties have been used commercially since 2005 (DGAV 2022). In Spain, it is possible to distinguish three different stages according to the level of adoption: 1) <10% of the total (grain and forage) maize cultivated in the country during 1998–2002; 2) between 10 and 20% from 2003 to 2010; and 3) between 20 and 30% from 2011 to 2021 (Fig. 2B) (MAPA 2022a, 2022b). The increase in adoption of Bt maize has been higher in regions where target pests cause severe and recurrent damage, as in Aragon, Catalonia, and Navarre in northeastern (NE) Spain (Fig. 2B and C). In Aragon, Bt maize adoption gradually increased from less than 30 to 70%, between 2003 and 2014; afterwards the adoption rate oscillated between 50 and 60% (Fig. 2B). The increase in use was steeper in Catalonia than in Aragon, rising from 40 to 70% between 2006 and 2008, and fluctuating around 70% since 2009 (Fig. 2B). Finally in Navarre, a slower increase in adoption rate has taken place, from less than 20% in 2004 to around 45% in 2016 and the following years (Fig. 2B). In Portugal, Bt maize is mostly grown in the Alentejo region and its adoption has remained below 25% (Fig. 2C) (DGAV 2022).

The main threat to the long-term success of insect-resistant GM crops is the evolution of resistance in target pests (Tabashnik et al. 2008, Tabashnik and Carrière 2019, Tabashnik et al. 2022). Therefore, since the approval of Bt maize cultivation in the EU, Insect Resistance Management (IRM) programs have been implemented to prevent or delay the evolution of resistance (European Commission 2001). These IRM programs include resistance monitoring to assess whether susceptibility of target pests to Cry1Ab maize may decline over time. Whereas a previous review focused on IRM for Bt maize in Spain, including data produced in our laboratory through 2019 and published without our consent (Álvarez-Alfageme et al. 2022), the present review updates this topic for the EU by including bioassay data from our laboratory through 2021 and addresses challenges and future prospects.

## Target Pests of Bt Maize in the EU

Insect pests are a major cause of economic losses in global maize production (Bradshaw et al. 2016, Manosathiyadevan et al. 2017), with several lepidopteran and coleopteran key pests limiting the yield of this crop (Edde 2022). In Europe, two of the most destructive pests of maize are the corn borers *S. nonagrioides* and *O. nubilalis*, which are the target pests of Cry1Ab maize. Larvae of both moths penetrate inside the maize stalks, where they excavate galleries and feed on the plant stem. This causes the stalks to break and the ears to fall, resulting in reduced yields. Stalk damage from both species is very similar (Eizaguirre and Fantinou 2012), but *S. nonagrioides* larvae are more voracious (Butrón et al. 1999, Velasco et al. 1999). Yield reductions also result from *O. nubilalis* larval feeding on kernels later in the season (Andreadis et al. 2008). In addition, damage from both corn borers promotes mycotoxigenic fungal growth, such as *Fusarium* spp. Link, which cause two of the most common forms of maize ear rot in Europe, the red ear rot and the pink ear rot (Logrieco et al. 2002). Mycotoxin contamination of maize grain is an important problem associated with the presence of corn borers and fungal growth, reducing crop quality and increasing the risks associated with food and feed production (Pitt et al. 2013, Arias-Martín et al. 2021). It has been estimated that between 2.25 and 4 million hectares of maize are directly affected annually by corn borers in Europe (Brookes 2019), causing annual average yield losses that range from 5 to 30% (Meissle et al. 2010). Knowledge about the biology and ecology of these target pests is essential to optimize the use of Bt maize and to design efficient IRM plans to avoid rapid evolution of resistance. However, there are still gaps in knowledge to be filled, especially in the case of *S. nonagrioides* (Castañera et al. 2016).

*S. nonagrioides* is distributed across the Mediterranean basin, including Southern Europe, the Middle East, and countries in North and Western Africa (Eizaguirre and Fantinou 2012), where it is one of the most damaging pests of maize (Anglade 1972, Melamed-Madjar and Tam 1980, Castañera 1986). It is a polyphagous species with host plants in the Poaceae, Cyperaceae, and Thyphaceae families (Kfir et al. 2002, Le Rü et al. 2006). Premating movement is rare among females and mating occurs near adult emergence sites, which might have important implications for Bt resistance management (Eizaguirre et al. 2004). After mating, females deposit eggs clutches between the leaf sheath and the stalk (López et al. 2003). First instar larvae begin to excavate galleries towards the interior of the plant, which makes larval control with conventional insecticides difficult (Velasco et al. 1999). Larvae usually molt 5 times during development (López et al. 2001), reaching 3.5–4 cm in their last instar (Anglade 1972). Movement among plants has been reported for last instar larvae (Eizaguirre et al. 2004). The number of generations per year depends on the climatic zone, latitude, and temperature. Two to three generations have been recorded in Spain and France (Anglade 1972, Galichet 1982, Eizaguirre et al. 2002) and up to four in Morocco and Portugal (Hilal 1977, Figueiredo and Araújo 1996).

*O. nubilalis* currently occurs in Europe, North Africa, West Asia, and North America. It is a highly polyphagous species feeding on more than 200 wild plants. It colonized maize after this crop was introduced to Europe about 500 years ago (Gould 1998, Marçon et al. 1999a, Bethenod et al. 2005). Adults can disperse tens of km in just a few nights (Showers et al. 2001). After emergence some adults move locally but others enter long-range dispersal (Bourguet et al. 2000a, b, Bailey et al. 2007, Gaspers 2009). Predispersal mating likely occurs for both males and females (Dalecky et al. 2006, Dorhout et al. 2008, Hu and Andow 2011). Females lay clutches of eggs on the underside of leaves and early larval instars feed on developing leaves and whorl tissues (Gaspers 2009). Larvae do not penetrate the stalk until the beginning of the third instar, and once inside feed on the meristematic tissue of the stem, digging galleries in the stalk (Mason et al. 1996, Mencarelli et al. 2013). In Europe, univoltine populations have been reported in Germany and the Netherlands, whereas in warmer regions of southern Europe, multivoltine populations are present (Gaspers 2009, Keszthelyi 2010). Two pheromone races (E- and Z-) have been identified: the E-race is found in Switzerland, Italy and Eastern North America; and the Z-race predominates over most of Europe and North America (Marçon et al. 1999a). Both races are present in Spain, although the Z-race is predominant (Gaspers 2009). Interestingly, a study in the USA has shown that susceptibility of this species to Cry1Ab does not differ between pheromone races, voltinism ecotypes or geographical areas (Marçon et al. 1999b).

Other noctuid moths are occasional pests of maize in Europe but are not considered target pests of Cry1Ab maize. Two of the most important are *Mythimna unipuncta* (Haworth), which can cause severe defoliation and *Helicoverpa armigera* (Hübner), which can damage silk and cob tips (Pérez-Hedo et al. 2012). Both species have low susceptibility to Cry1Ab (Eizaguirre et al. 2010, González-Cabrera et al. 2013, Pérez-Hedo et al. 2013).

## Insect Resistance Management for Bt Maize in the EU

Since commercialization of Bt maize in the EU, IRM plans have been implemented to prevent or delay the evolution of resistance to Cry1Ab (Head and Greenplate 2012). IRM plans are based on the high-dose/refuge (HDR) strategy, which is commonly used worldwide and mandatory in the EU (EFSA 2018). This strategy is based on cultivation of Bt crops that produce a high concentration of insecticidal proteins effective against the targeted pests and the deployment of refuges (non-Bt crop host plants) near Bt crops (Siegfried and Hellmich 2012, Tabashnik et al. 2013). In many countries this strategy is currently based on use of pyramided Bt crops that produce more than one toxin effective against the same target pest (Bates et al. 2005, Carriére et al. 2015, 2016, Van den Berg et al. 2022). However, this strategy cannot be implemented in the EU since only MON810 varieties that produce Cry1Ab are approved for cultivation (European Commission 2022a). An important part of IRM plans is the implementation of a monitoring program that enables early detection of resistance evolution, which may allow the application of corrective measures in a timely manner if resistant populations are detected (Andow and Ives 2002, Head and Greenplate 2012). All European Bt maize is concentrated in the Iberian Peninsula, mostly in Spain. Thus, the EU resistance monitoring plans for pests targeted by Bt maize have focused primarily on this country (Farinós et al. 2018, Thieme et al. 2018, Bertho et al. 2020), particularly northeast Spain since 2016 (EFSA 2017).

Success of the HDR strategy used to delay evolution of resistance to single-toxin Bt crops can be improved by several conditions, including: 1) recessive inheritance of resistance; 2) most matings between the rare individuals that survive on the Bt crop should be with susceptible individuals from refuges; and 3) resistance allele frequencies in field populations should be low (Roush 1994, Bates et al. 2005, Tabashnik et al. 2013). Violations of any of these conditions can increase the likelihood of resistance evolution (Gassmann et al. 2011, Tabashnik et al. 2013, Tabashnik and Carrière 2019, Huang 2021).

Bt plants should produce a sufficient concentration of toxin to kill heterozygote individuals carrying a single resistance allele (Carrière et al. 2010). As a consequence, only homozygous resistant individuals would survive on Bt plants, and mate with the more abundant susceptible individuals produced in refuges (Andow 2008). Their offspring would be heterozygote, and thus susceptible to Bt plants (Bourguet et al. 2000c). Maize varieties carrying the event MON810, which are the only GM varieties grown in Europe, produce high levels of Cry1Ab throughout their development (Székács et al. 2010a,b), which is enough to kill 100% of susceptible larvae of both *O. nubilalis* and *S. nonagrioides* (Farinós et al. 2018). Thus, it is assumed that they fulfill the high dose requirement, though no Cry1Ab resistant populations of *O. nubilalis* nor *S. nonagrioides* have been detected to determine if resistance in these species is effectively recessive. By contrast, maize hybrids derived from event Bt176 cultivated between 1998 and 2005 showed a decrease in toxin concentration during plant development (Fearing et al. 1997). This resulted in larvae being exposed to lower concentrations of the toxin, which could have favored the evolution of resistance (Archer et al. 2000, Magg et al. 2001).

Both in Portugal and Spain, mandated refuges must be at least 20% of the area planted with Bt maize. Conventional maize that has a similar phenology as Bt maize should be used in refuges, since maize is the main host of both *O. nubilalis* and *S. nonagrioides* (Bourguet et al. 2000b, Camargo et al. 2020). Refuges can be sown in the same field or in adjacent fields at a maximum distance of 750 m, because of limited dispersal of *S. nonagrioides* short flight patterns. In Spain, refuges are not required for growers who plant less than 5 ha of Bt maize. In Portugal, planting refuges is always mandatory. Refuge compliance in Spain has been above 80% since 2008 and above 90% since 2015, while in Portugal it has been 100% according to questionnaires sent to growers (Farinós et al. 2018, European Commission 2022b).

The frequency of alleles conferring resistance to MON810 maize was estimated in EU field populations of *O. nubilalis* and *S. nonagrioides* between 2003 and 2005. Resistant allele frequencies were 0.0001 (CI 95% 0.0–3.0 × 10^−4^) for *O. nubilalis* (Engels et al. 2010) and 0.0015 (CI 95% 0.0–4.6 × 10^−3^) for *S. nonagrioides* (Andreadis et al. 2007).

## Resistance Monitoring Plans for Bt Maize in the EU

Monitoring plans in the EU aim to detect declines in susceptibility of the targeted pests to Cry1Ab in areas where Bt maize is grown. These plans are mandatory since 2001, when the European Parliament Directive 2001/18/CE of March 12th was published (European Commission 2001). However, the Spanish Central Administration started their own monitoring plan three years before this directive and this was implemented until 2011 (MITECO 2010). Complementary to this effort, seed companies have had their own monitoring plan since 2004 (Farinós et al. 2018, Thieme et al. 2018). The EU monitoring plans have focused mainly on the Iberian Peninsula (Spain and Portugal) where adoption of Bt maize has been higher. Monitoring was also carried out in some years between 2000 and 2010 in southwestern France (Midi-Pyrénées and Poitout-Charentes) for *S. nonagrioides* and in the Czech Republic, France, Germany, Italy, Hungary, Slovakia, Poland, and Romania for *O. nubilalis* (Farinós et al. 2018, Thieme et al. 2018).

Results of the monitoring performed by the Spanish Administration between 1999 and 2011 and by seed companies between 2004 and 2015 have been published (Farinós et al. 2004, 2011, 2018; Castañera et al. 2016, Thieme et al. 2018, Bertho et al. 2020). In Spain and Portugal they focused on the northeast (Ebro Valley, Spain), center (Madrid and Castilla-La Mancha, Spain), and southwest (Alentejo, Portugal; and Extremadura and Andalusia, Spain) (Fig. 2C). Sampling in these areas was carried out every other year. Susceptibility assessment relied on concentration-response diet-overlay bioassays with purified Cry1Ab to estimate lethal concentrations (LC_50_) or molt inhibition concentrations (MIC_50_) affecting 50% of the population. The results indicated no shifts over time in the susceptibility of *S. nonagrioides* or *O. nubilalis* to Cry1Ab. In France, where MON810 hybrids were cultivated in 2005–2007, *S. nonagrioides* variation in both MIC_50_ and LC_50_ was in the range observed for Iberian populations (Farinós et al. 2018). Likewise, only small variation in susceptibility (MIC_50_) to Cry1Ab was observed for European *O. nubilalis* populations from other geographic regions (Thieme et al. 2018). 

### Update on the Status of the Monitoring Program

**Fig. 3.**
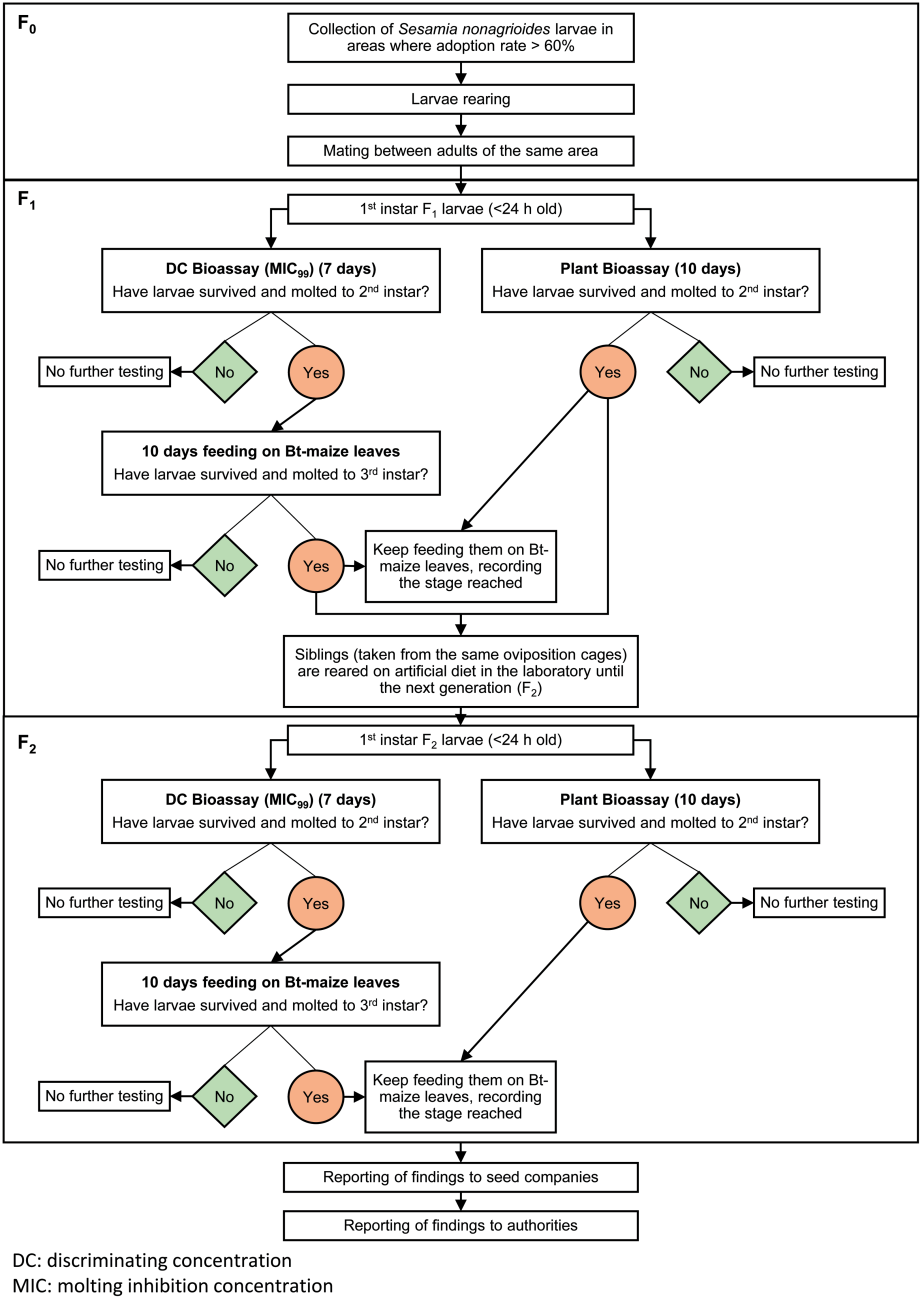
Flowchart of the monitoring strategy for *S. nonagrioides* resistance to MON810 maize in the EU.

In 2016, the monitoring plan changed in accordance with the data gathered for more than a decade and the recommendations of the European Food Safety Authority (EFSA 2015, 2016a). Since then, a stepwise approach has been followed (Fig. 3, approach for *S. nonagrioides*). The aims of the changes were to strengthen the efforts in areas where resistance is more likely to evolve and to reach a minimum detection threshold for resistance allele frequency of 3%. Thus, insect collection has been focused on hotspots, i.e., areas where adoption rate of Bt maize surpasses 60% and targeted pests have two or more generations yearly (EFSA 2015, 2016a). In the EU, this situation only occurs in NE Spain, where monitoring is now carried out annually. Diagnostic concentration (DC) bioassays with Cry1Ab, intended to cause ≥99% molting inhibition for first instar larvae (MIC_99_), and plant bioassays with Cry1Ab maize are used to measure susceptibility of both species. The bioassays for *S. nonagrioides* are performed by our group whereas the laboratory BTL GmbH Sagerheide (Germany) carries out bioassays for *O. nubilalis*. An annual report is sent by the seed companies to EFSA for evaluation, revision, and modification when necessary. These reports have been available since 2009 at the EC website (European Commission 2022b).

**Fig. 4.**
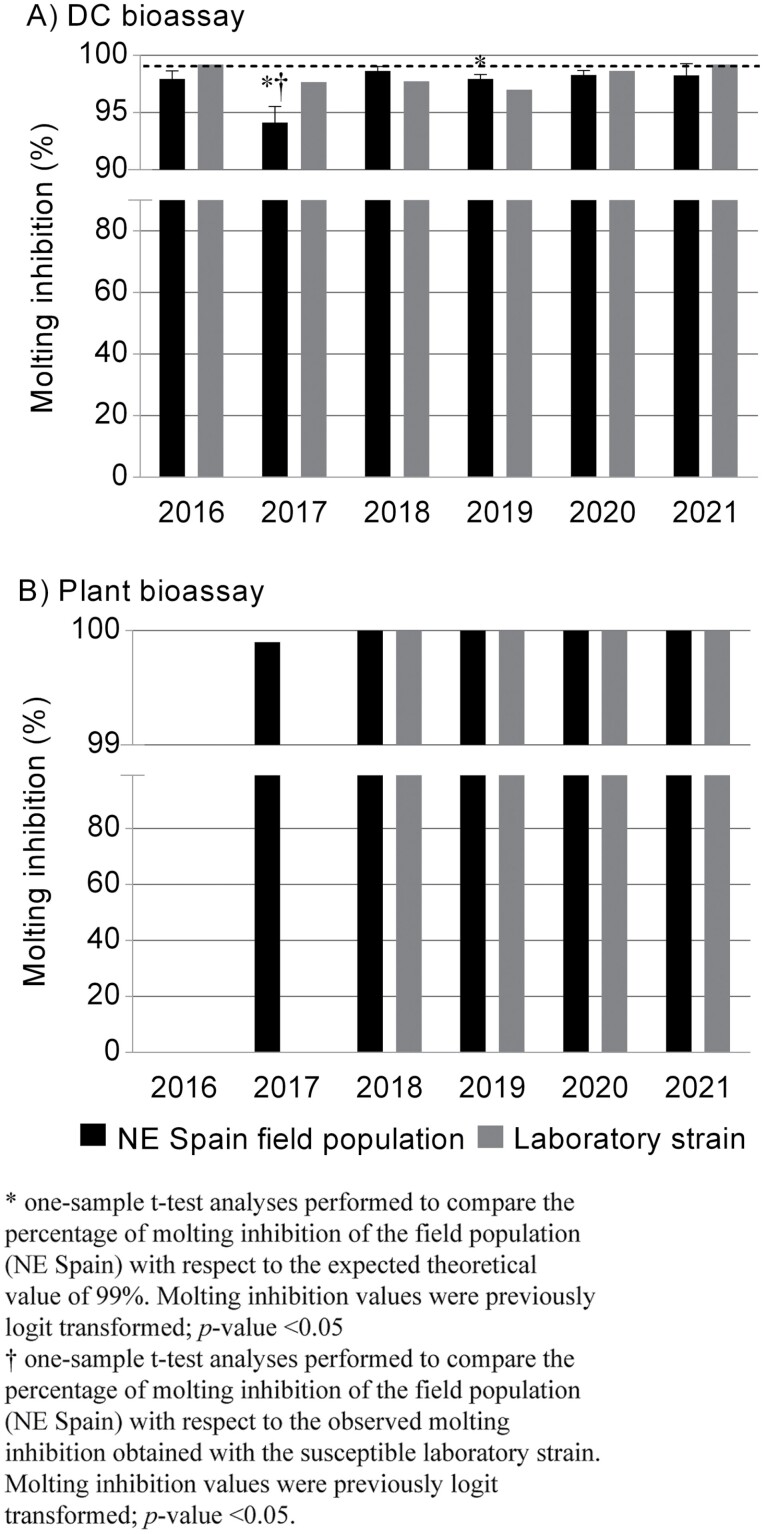
Molting inhibition of *S. nonagrioides* larvae coming from NE Spain field populations (black columns) or from a susceptible laboratory strain (grey columns) from 2016 to 2021. (A) Diagnostic concentration (DC) bioassays using Cry1Ab, intended to cause ≥99% molting inhibition to first-instar larvae (MIC99, dash line), (B) Plant bioassays with Bt maize. Source: https://food.ec.europa.eu/plants/genetically-modified-organisms/post-authorisation/monitoring-plans-and-reports_en#pmem-reports-for-gm-food-and-feed

Between 2016 and 2021 no significant shifts were observed in the susceptibility of NE Spain field populations of *S. nonagrioides* to Cry1Ab in DC bioassays (Fig. 4A) or plant bioassays (Fig. 4B). During the DC bioassays the susceptibility of the field-collected larvae is checked against both a theoretical molt inhibition value of 99% and the molt inhibition displayed by the reference strain. Statistically significant differences were detected in 2017 and 2019 between the observed and the expected MIC_99_ values obtained in DC bioassays (Fig. 4A). In 2017 the observed MIC_99_ value of the field populations was significantly lower than that of the laboratory strain (Fig. 4A). In plant bioassays, the mortality of first instar larvae from field collected populations was 99.9% in 2017, the first year this assay was performed, and 100% in subsequent years (Fig. 4B). Throughout these years, no clear trends are observed, suggesting that the differences noticed between some of the years could be attributable to natural variation in the field populations. Likewise, no decrease in the susceptibility to Cry1Ab has been detected in *O. nubilalis* (European Commission 2022b). These results confirm that MON810 maize is still effective against both species and there are no signs of field evolved resistance.

### Assessment of the Implementation of the Current Monitoring Plan

A critical reassessment of the monitoring plan carried out in 2004–2015 has already been accomplished (Farinós et al. 2018). Recommendations implemented since 2016 (EFSA 2015) include focusing on target pest populations from hotspots and reducing the detection limit for resistance allele frequency from 5 to near 3%. However, the experience accumulated through the implementation of this new monitoring plan has revealed some practical and technical limitations, which are addressed below.

#### Sources of Agronomic Data

**Fig. 5.**
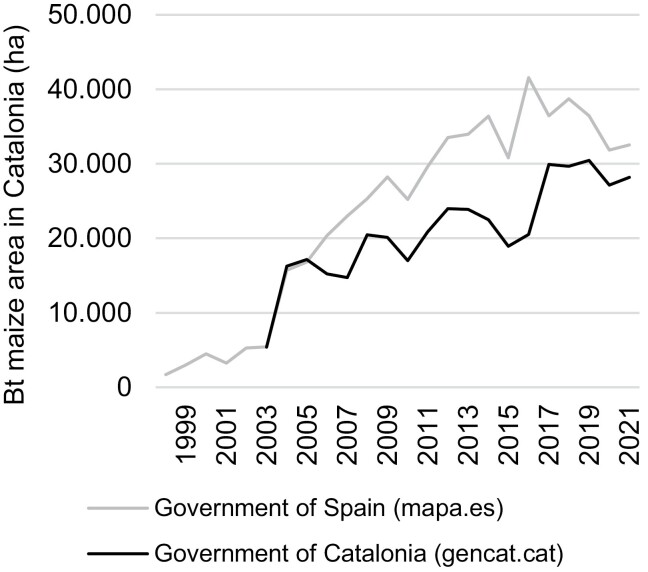
Differences in the Bt maize area reported by different Spanish authorities (MAPA 2022a, GENCAT 2022).

The use of reliable and thorough agronomic databases is essential to identify areas with the greatest selection pressure to be incorporated in resistance monitoring plans. However, in Spain, there are notable differences in the estimated areas of Bt maize cultivated annually, depending on the administration that generates these data. The acreage data provided by the Spanish Ministry of Agriculture is an indirect measure obtained from the number of seeds sold, provided by the companies, and the maize planting density. However, taking as an example Catalonia, one of the regions with the highest level of adoption of Bt maize, data on cultivated areas are also provided by the local Government based on questionnaires answered by farmers (Fig. 5). This may give rise to inconsistencies between estimates provided by the Spanish Ministry of Agriculture and local Governments that complicate decision making. Other data needed for the monitoring plan, such as the area of Bt maize dedicated to grain or feed maize, or first or second harvest maize, which varies depending on regions, are sometimes unavailable or difficult to access.

#### Insect Collection

Last-instar larvae of *S. nonagrioides* and *O. nubilalis* are collected for bioassays following standard operative procedures (EuropaBio 2017), from three sampling zones in NE Spain each year, each with a maximum 10 km diameter. The goal is to collect a minimum of 1,000 larvae of each species since, to achieve a 3% detection limit for resistant alleles, this is the number required for larval screens assuming recessive alleles (1/*N*^1/2^, where *N* is the number of larvae) (Andow and Ives 2002). The protocol establishes the collection of at least 350 larvae from three fields, with a minimum of 50 larvae per field (successful field) within each sampling zone. This objective has been met for *S. nonagrioides* for each campaign (Table 1). However, since 2016 it has proven difficult to meet this requirement for *O. nubilalis*, and it has not been possible in the last two campaigns (Table 1). Additionally, the targeted number of successful fields (three in each zone) has not been met in any of the campaigns for *O. nubilalis* even though the number of prospected fields has increased annually (Table 1).

**Table 1.** Collection of *S. nonagrioides* and *O. nubilalis* larvae and resistance allele detection limit for *S. nonagrioides*, corresponding to the European MON810 maize resistance monitoring plan carried out from 2016 to 2021

		2016	2017	2018	2019	2020	2021
*S. nonagrioides*	Larvae collected	1,364	1,452	1,490	1,644	1,590	1,699
	Prospected fields	29	19	18	28	27	24
	Successful fields^*a*^	11	9	9	9	11	12
	Larvae per field	47.0	76.4	82.8	58.7	58.9	70.8
	Adults represented in bioassays^*b*^	911 (67%)	749 (52%)	554 (37%)	868 (53%)	787 (50%)	1076 (63%)
	Resistance allele detection limit	3.3%	3.7%	4.3%	3.4%	3.6%	3.0%
*O. nubilalis*	Larvae collected	1,111	1,111	1,144	1,110	651	811
	Prospected fields	17	19	23	28	27	29
	Successful fields^*a*^	6	5	7	8	4	5
	Larvae per field	65.4	58.5	49.7	39.6	24.1	28.0

^*a*^Successful fields are those where at least 50 larvae were collected.

^*b*^Number of adults, reared from larvae collected in the field, whose progeny were used in the bioassays. The percentage with respect to the total number of larvae is indicated among brackets.

The difficulties encountered in collecting enough *O. nubilalis* larvae could be related to regional pest suppression due to continued Bt maize cultivation in NE Spain since 1998. Regional pest suppression through high adoption of Bt maize has been reported for *O. nubilalis* in the United States (Hutchison et al. 2010, Bohnenblust et al. 2014) and for several other pests (e.g., Carrière et al. 2003, Wu et al. 2008). Such regional declines may provide important agronomic benefits, for farmers growing Bt maize, and other types on non-Bt crops (Hutchison et al. 2010, Dively et al. 2018). However, the lack of available historical records of damage caused by the corn borer or population density of this pest in Spain does not allow us to verify that regional declines have occurred. Additional studies are needed to determine if declines in the densities of this pest have occurred in areas where they have historically caused important damage to maize.

#### Insect Rearing

Diapausing larvae of both species are usually collected, and kept in this condition until they emerge from diapause. They are then reared until pupation and subsequent adult emergence for mating. Some of the individuals collected are lost during rearing, mainly in the larval stage, due to pathogens or parasitoids (mostly Tachinidae in the case of *S. nonagrioides*), although there is also mortality due to malformations in pupae and adults (Supp Fig. 1 [online only]). This mortality, together with the infertility of some adults, means that not all individuals collected in the field are represented in bioassays (Table 1).

*S. nonagrioides* preimaginal mortality for larvae collected in the period 2016–2021 was 42 ± 5% with relatively high variability observed between years (from 30% in 2016 to 61% in 2018). Such larval mortality has hindered the resistance allele detection limit, only reaching the target 3% in the 2021 campaign, even though the number of larvae collected had increased over the years (Table 1). Optimization of several procedures to reduce this preimaginal mortality have been tried and implemented, such as conducting larvae disinfection, reduction in the number of larvae per rearing box, reduction in the number of times per week that the rearing boxes are checked to avoid excessive handling and increasing the change of vermiculite during the diapause period, among others. However, no significant improvements in preimaginal survival rates have been observed.

Similar problems have been experienced by the BTL GmbH Sagerheide laboratory that assesses the susceptibility of *O. nubilalis* field populations collected in Spain, which has not been able to reduce the allelic detection limit for this species below 4% (European Commission 2022b).

#### Laboratory Strains

**Fig. 6.**
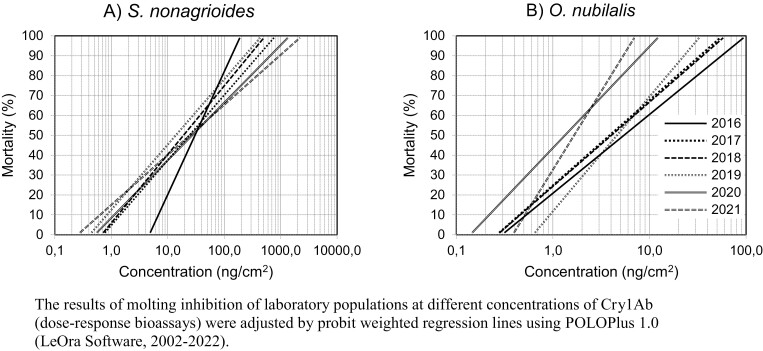
Regression lines of mortality for laboratory strains of *S. nonagrioides* (A) and *O. nubilalis* (B) between 2016 and 2021. Source: https://food.ec.europa.eu/plants/genetically-modified-organisms/post-authorisation/monitoring-plans-and-reports_en#pmem-reports-for-gm-food-and-feed

It has proven difficult to maintain reference strains of some lepidopteran species for many years in the laboratory, mainly because they suffer excessive inbreeding (Roush 1986), but also due to pathogens accidentally introduced in the laboratory by field collected populations. To tackle this issue, the reference strains of *S. nonagrioides* and *O. nubilalis* are refreshed periodically with specimens collected in non-Bt fields from Galicia (northwestern Spain), where Bt maize has never been commercially grown. Even so, the performance of laboratory strains can still decline over time, as the strains often demonstrate decreasing larval weight and adult fertility. In those cases, new reference strains are generated with individuals collected in Galicia, taking some precautions: 1) comparison of the LC_50_ values of both laboratory (Fig. 6) and field populations, estimated by concentration-response diet-overlay bioassays with purified Cry1Ab (Farinós et al. 2018), to guarantee that there are no significant differences between them; 2) check for presence of pathogens (namely *Nosema* sp.) by microscopy and by molecular methods (PCR); and 3) ensure that the new population is adapted to feeding on an artificial diet under laboratory conditions.

## Why has Resistance not Evolved? Sustained Efficacy of Bt Maize against *S. nonagrioides*

The evolution of resistance of field populations to Bt maize producing Cry toxins has occurred in at least six lepidopteran species to date. Four of them are noctuids (Lepidoptera: Noctuidae): *Busseola fusca* (Fuller) in South Africa, the fall armyworm *Spodoptera frugiperda* (JE Smith) in the United States, Brazil, and Argentina, *Helicoverpa zea* (Boddie), in the United States and *Striacosta albicosta* Smith in the United States and Canada; and two crambids (Lepidoptera: Crambidae): *Diatraea saccharalis* (Fabricius) in Argentina and *O. nubilalis* in Canada (Smith et al. 2019, Tabashnik and Carrière 2019, Tabashnik et al. 2022). Such field-evolved resistance has been attributed to a variety of causes, including exposure of the larvae to low concentrations of the toxin (*B. fusca*, *H. zea*, *S. frugiperda*, and *S. albicosta*) which may yield nonrecessive inheritance of resistance (Tabashnik et al. 2013), high adoption rate and repeated cultivation (*B. fusca*, *H. zea*, *S. frugiperda*, *S. albicosta*, *D. saccharalis*, and *O. nubilalis*), low refuge compliance (*B. fusca*, *H. zea*, *S. frugiperda*, *D. saccharalis*, and *O. nubilalis*), and lack of long-range migration from outside agricultural systems due to geographic or agronomic characteristics, which limits genetic variability (*B. fusca*, *H. zea*, and *S. frugiperda*) (Van Rensburg 2007, Dively et al. 2016, Omoto et al. 2016, Ostrem et al. 2016, Chandrasena et al. 2017, Smith et al. 2017, Campagne et al. 2013, Grimi et al. 2018, Smith et al. 2019).

The corn borer *S*. *nonagrioides* presents certain biological characteristics that could accelerate the evolution of resistance to Bt maize: 1) despite being a polyphagous species, it is considered oligophagous or largely monophagous in maize areas (Castañera et al. 2016, Camargo et al. 2020); 2) multivoltine populations may have increased exposure to Bt maize (Eizaguirre et al. 2002); 3) adults, especially those of the third generation, have low dispersal between refuges and Bt maize (Eizaguirre et al. 2006) and there is a low genetic exchange between distant populations (De La Poza et al. 2008); and 4) females mate before they disperse to oviposit (Lopez et al. 1999), so adults from refuges would rarely mate with the moths that eventually emerge from Bt maize. There are also a number of agronomic characteristics associated with the cultivation of Bt maize in NE Spain that could also accelerate evolution of resistance in this area, such as high adoption rate of Bt maize in the region and continuous cultivation of Bt maize. Altogether, the biological and agronomic traits define the region as a resistance hotspot for *S. nonagrioides* (EFSA 2018). However, no resistant populations have been detected (European Commission 2022b).

To determine what has delayed the evolution of resistance of *S. nonagrioides* to Cry1Ab maize, a resistance evolution model was developed (Castañera et al. 2016). The model includes three different types of parameters: 1) population parameters (proportion of adults leaving fields; relative preference for Bt and non-Bt fields; adult fecundity and survival; diapause rate and overwintering survival); 2) genetic parameters (initial R allele frequency; RR, RS and SS survival on each type of maize and in each generation; assortative mating in each generation); and 3) environmental and agronomical parameters for the period 1998 and 2013 (proportion of each type of maize, i.e., Bt maize (event Bt176 or MON810) versus non-Bt maize; proportion of maize rotated to another crop; refuge compliance). This model predicted that, if current cultivation conditions continue, the resistance allele frequency would be >0.5 by 2050 in the Ebro Valley (Castañera et al. 2016). The modeling results indicate withdrawal of Bt176 from the market was a critical factor in delaying the evolution of resistance because of the low concentration of Cry1Ab late in the season in this event. They also emphasized the importance of compliance with the planting of refuges in areas with high adoption rate of Bt maize such as the Ebro Valley, and for refuges to be located close to Bt maize fields to enhance mating between resistant and susceptible adults. Currently, the guide of good practices for planting Bt maize in Spain has established this distance at <750 m (MAPA 2015, European Commission 2022b).

The F_2_ screen method (Andow and Alstad 1998) was used twice to determine the frequency of resistance alleles in field populations of *S. nonagrioides* from southern Europe: 1) in 2004–2005 in NE Spain (Ebro Valley), after 7–8 years of Bt maize cultivation and with an adoption rate around 30%, and in Greece, where Bt maize has never been grown; and 2) in 2016, again in the Ebro Valley, after 18 years of cultivation and with a mean adoption rate of 64%. The frequency of resistance alleles in the southern European populations was estimated as 0.0015 (0.0032 for Greece and 0.0029 for Ebro Valley) in 2004–2005 (Andreadis et al. 2007). These results reflect the absence of isolines giving positive results (survivors at F_2_) although only 85 isolines were tested from the Ebro Valley. In 2016, 1 of 137 isolines was scored as positive for resistance, yielding an estimated resistance allele frequency of 0.0036 (Camargo et al. 2018). This new value was used to update the existing model (Andreadis et al. 2007). The results indicate that resistance would occur in 31 years from 2016, 2.8 years earlier than expected with the allelic frequency of 0.0029 estimated in 2004–2005 (Castañera et al. 2016, Camargo et al. 2018). The estimated resistance allele frequency was not significantly higher in 2016 than 2004–2005. The monitoring and modeling results imply the HDR strategy could be effective in delaying the evolution of resistance in the NE Spain if strict compliance with the refuge requirements continues.

Gaps that need to be filled to confirm key assumptions and further refine model results are mainly related with pest’s biology (assortative mating and use of other hosts at certain times of the year or in certain zones, etc.) (Castañera et al 2016). Moreover, global warming may change some factors such as the length of the crop season.

## Challenges and Future Prospects

The year 2022 is the 25th year of continuous planting of Bt maize in Spain, and the 19th year since planting of varieties derived from the event MON810 began. To date, the monitoring data show that the target pests *S. nonagrioides* and *O. nubilalis* have not evolved resistance to Cry1Ab in the field. Also, seed marketing companies have not received reports of confirmed control failures (European Commission 2022b). The efficacy of MON810 in controlling these pests and compliance with the mandatory refuge planting in resistance hotspots, especially during the last fifteen years (both the companies marketing GM seeds and the competent authorities have made great informational efforts) may have contributed to this success. However, the EU is facing several challenges that may jeopardize the planting of GM crops in the future.

The main challenge for the cultivation of Bt maize in the EU is social rejection in many countries. As a consequence, new events cannot be readily approved in the EU, despite their potential agronomic, economic, social, and environmental benefits (Gomez-Barbero et al. 2008, Brookes 2019, Areal and Riesgo 2022). New authorizations of GMOs require approval by a qualified majority of Member States in the Council of the EU, yet many countries oppose cultivation of GMOs (Tagliabue 2017, Hundleby and Harwood 2019). This situation is not likely to change soon (Eriksson et al. 2019). Also, in some situations, farmers face economic losses or increased labor to comply with coexistence regulations, i.e., legal requirements imposed by the European Commission to ensure GM crops are not mixed with non-GM crops at the farm level (European Commission 2010 , Park et al. 2011, Kathage et al. 2016).

The limitations in the EU for the approval of new GM crops are a serious constraint for improving resistance management strategies, because pyramided Bt maize hybrids producing several Bt toxins are not allowed. The simultaneous use of multiple toxins that do not exhibit cross-resistance is one of the main tactics recommended for delaying insect resistance to Bt crops (Roush 1998, Carrière et al. 2015, 2016). This approach is based on the fact that if resistance to each toxin in a pyramid is rare, individuals with resistance to all toxins will be extremely rare (Tabashnik 1994). A large number of pyramided Bt maize hybrids are available (ISAAA 2022), which can improve control of maize pests and resistance management (Carrière et al. 2015, 2016; Marques et al. 2019). Pyramids have strong potential to enhance resistance management for *O. nubilalis* (Tan et al. 2013) and would likely be useful to improve management of resistance for *S. nonagrioides*, since laboratory studies show high susceptibility of this species to Cry1Ab and Cry1Fa and potentially low cross-resistance between these toxins (González-Cabrera et al. 2006).

The current situation in the EU regarding the difficulty of approving new GM crops is also constraining control of invasive and emerging key pests of maize, such as the Western corn rootworm, *Diabrotica virgifera virgifera* LeConte (Coleoptera: Chrysomelidae). The advance in Europe of *D. v. virgifera* is a matter of growing concern. Originally from Central America, this species now occurs in more than 15 European countries, including France, Italy, Greece, Poland, Czech Republic, Hungary, Romania, and Croatia (Mrganić et al. 2018, Bažok et al. 2021). *Diabrotica v. virgifera* has also been recently reported in Spain in Aragon and Catalonia in the NE of the country (Mateu et al. 2021, CSCV 2021). In the United States where *D. v. virgifera* is a major pest of maize (Gassmann et al. 2011), one of the main tools for its control has been pyramided Bt maize that will be combined with use of RNA interference (RNAi) starting in 2022 (Carrière et al. 2020, Meinke et al. 2021). In Europe, the level of damage caused by this pest varies depending on the area and its control is based on crop rotation, as the cultivation of Bt maize varieties effective against this coleopteran pest is not authorized (Bažok et al. 2021). However, this beetle has so far overcome different management practices, including crop rotation, conventional insecticides, and Bt maize (Wright et al. 2000, Levine et al. 2002, Carrière et al. 2020, Gassmann 2021). Since 1995, monitoring by European countries has measured population fluctuations of *D. v. virgifera* (Bažok et al. 2021).

There is also concern that *S. frugiperda* may become established in continental Europe. Because this pest has low susceptibility to Cry1Ab, MON810 would not provide efficient control (Storer et al. 2010, Omoto et al. 2016). This key pest of maize has already evolved resistance to different Bt toxins in several populations in the Americas (Tabashnik and Carrière 2019, Tabashnik et al. 2022). This pest is a highly migratory species that has recently colonized most countries south of the Sahara and Southeast Asia, including India, and China, from where it has reached Australia (Goergen et al. 2016, EPPO 2022). It has also been recently introduced into the Canary Islands (Spain) (Moreno and Gastón 2020) from the African continent (EPPO 2021). Some forecasting models predict that southern and central Europe could be exposed to the risk of transient populations, especially with global warming (Tepa-Yotto et al. 2021, Gilioli et al. 2022). Also, there has been an increasing number of interceptions (33 in 2021 alone) of this species in plants and plant products imported into the EU from both the Americas and Africa (EUROPHYT 2022).

A recent issue of concern in the EU is the expansion of teosinte, the closest wild relative of maize. This invasive species was reported as a new agricultural weed in France in 1990 and in Spain in 2014. Its distribution has increased despite efforts to contain its advance (Pardo et al. 2016, Le Corre et al. 2020). Different agronomic and environmental consequences of hybridization between maize and teosinte have been identified, including the transfer of the *cry1Ab* transgene from Bt maize to teosinte, which could expose corn borers to low concentrations of the toxin and accelerate the evolution of resistance. The presence of the *cry1Ab* transgene has been demonstrated in different genetic backgrounds derived from Bt maize and teosinte crosses, giving rise to plants producing similar Cry1Ab concentrations as in the Bt parental hybrid (Erasmus et al. 2019; Lohn et al. 2021). However, mortality of *O. nubilalis* was sometimes less than 99% (Lohn et al. 2021). Although some uncertainties remain (EFSA 2022), EFSA (2016b) concluded there is no need to revise current risk management recommendations for Bt maize. However, further studies are warranted to assess the potential impact of teosinte on evolution of resistance to Bt maize in corn borers. These studies could evaluate factors such as the frequency of hybridization under field conditions with Bt maize varieties, the suitability of teosinte and hybrid progeny as host plants for corn borers and other nontarget lepidopterans, and the concentration of Cry1Ab in teosinte and hybrid progeny after several generations of crossing.

## Supplementary Material

toac154_suppl_Supplementary_Figure_S1
