# Supplementary material for: Monitoring Insect Resistance to Bt Maize in the European Union: Update, Challenges, and Future Prospects
Source: J Econ Entomol. 2023 Jan 4;116(2):275–88. doi: 10.1093/jee/toac154 (PMC10125040; doi:10.1093/jee/toac154)

Supplementary Figure 1. Daily evolution (percentage of the population) of field-collected *S. nonagrioides* larvae to the adult stage, reared under laboratory conditions. Larvae were collected in 3 different zones of northeastern Spain in 2020 and 2021, and most of them were in diapause at that time.

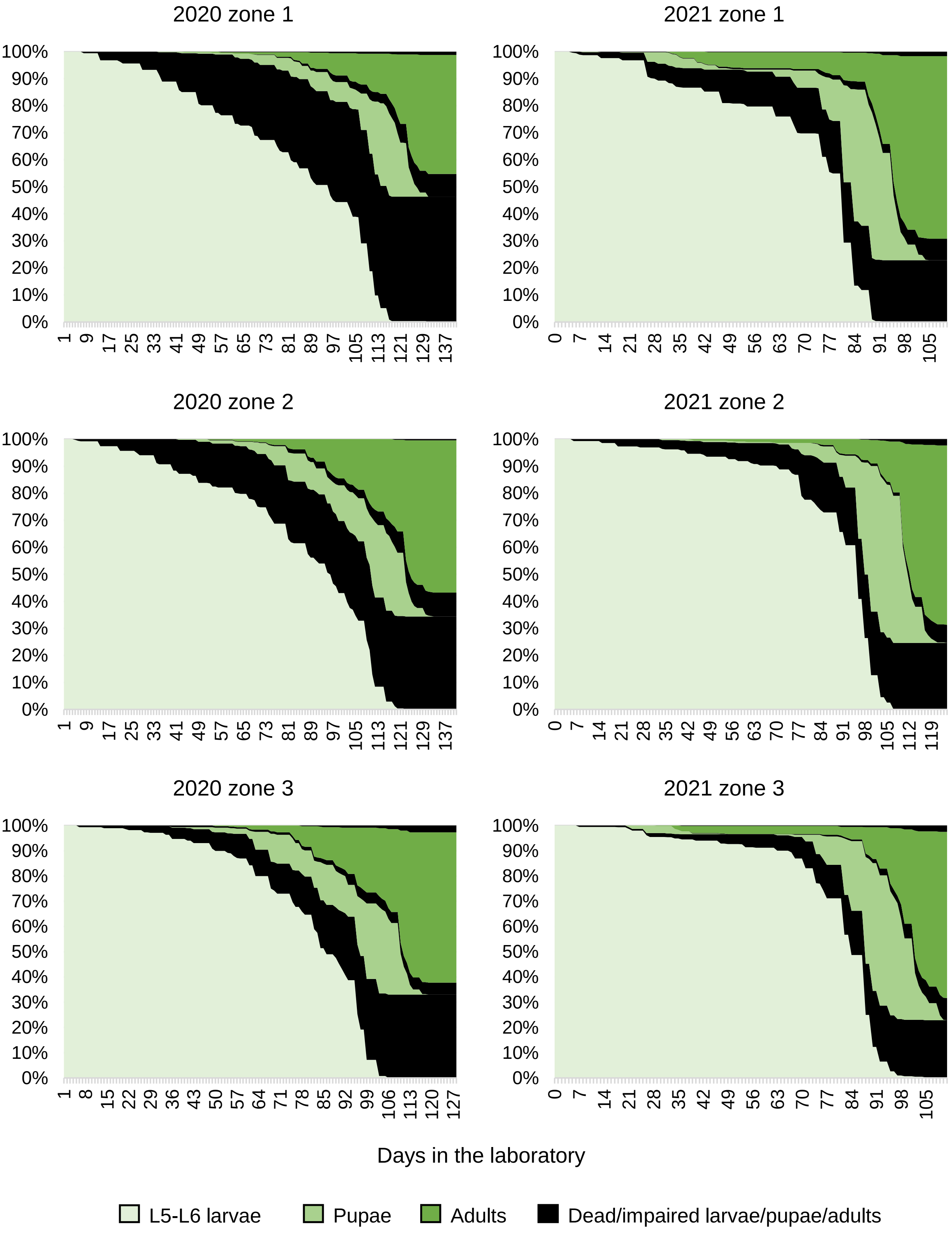

Supplement: toac154_suppl_Supplementary_Figure_S1 [file toac154_suppl_supplementary_figure_s1.pdf]
